# A genome-wide association study of red-blood cell fatty acids and ratios incorporating dietary covariates: Framingham Heart Study Offspring Cohort

**DOI:** 10.1371/journal.pone.0194882

**Published:** 2018-04-13

**Authors:** Anya Kalsbeek, Jenna Veenstra, Jason Westra, Craig Disselkoen, Kristin Koch, Katelyn A. McKenzie, Jacob O’Bott, Jason Vander Woude, Karen Fischer, Greg C. Shearer, William S. Harris, Nathan L. Tintle

**Affiliations:** 1 Department of Mathematics, Statistics and Computer Science, Dordt College, Sioux Center, Iowa, United States of America; 2 Department of Statistics, Baylor University, Waco, TX, United States of America; 3 Department of Statistics, Duke University, Durham, NC, United States of America; 4 Department of Mathematics and Statistics, University of Maryland- Baltimore County, Baltimore, MD, United States of America; 5 Department of Nutritional Sciences, Penn State University, State College, PA, United States of America; 6 OmegaQuant, Sioux Falls, SD, United States of America; University Hospital Jena, GERMANY

## Abstract

Recent analyses have suggested a strong heritable component to circulating fatty acid (FA) levels; however, only a limited number of genes have been identified which associate with FA levels. In order to expand upon a previous genome wide association study done on participants in the Framingham Heart Study Offspring Cohort and FA levels, we used data from 2,400 of these individuals for whom red blood cell FA profiles, dietary information and genotypes are available, and then conducted a genome-wide evaluation of potential genetic variants associated with 22 FAs and 15 FA ratios, after adjusting for relevant dietary covariates. Our analysis found nine previously identified loci associated with FA levels (*FADS*, *ELOVL2*, *PCOLCE2*, *LPCAT3*, *AGPAT4*, *NTAN1/PDXDC1*, *PKD2L1*, *HBS1L/MYB and RAB3GAP1/MCM6*), while identifying four novel loci. The latter include an association between variants in *CALN1* (Chromosome 7) and eicosapentaenoic acid (EPA), *DHRS4L2* (Chromosome 14) and a FA ratio measuring delta-9-desaturase activity, as well as two loci associated with less well understood proteins. Thus, the inclusion of dietary covariates had a modest impact, helping to uncover four additional loci. While genome-wide association studies continue to uncover additional genes associated with circulating FA levels, much of the heritable risk is yet to be explained, suggesting the potential role of rare genetic variation, epistasis and gene-environment interactions on FA levels as well. Further studies are needed to continue to understand the complex genetic picture of FA metabolism and synthesis.

## Introduction

Genome-wide association studies (GWAS) continue to be used in order to attempt to understand potential genetic contributions to phenotypes. Prior work has suggested a strong heritable component (24%) to fatty acid (FA) variation [1]. Recently, numerous studies have conducted genome-wide association analyses testing for associations with FA levels [2–5] identifying numerous loci. A common theme in these papers is a focus on the use of FA levels measured in the plasma.

In contrast, we recently conducted a GWAS using red-blood cell (RBC) FA measurements [6] on the Framingham Heart Study Offspring cohort. Plasma FA levels have been shown to be significantly impacted by recent change in diet, whereas RBC measurements have been shown to be more stable and thus may be a better indicator of chronic FA levels [7]. In our previous GWAS, we identified five loci associated with FA levels which reached genome-wide significance (*TRIM58*, *PCOLCE2*, *ELOVL2*, *FADS* genes and *LPCAT3*), three of which were new observations. However, this analysis was limited by only analyzing 14 FAs and no FA ratios, not considering dietary intake of FAs or fish oil supplements, considering only 2.5 million SNPs and only conducting single-marker tests of variant-FA association.

In this report, we have expanded our analysis to include 8 additional FAs and 15 biologically relevant FA ratios [8]; these ratios are used to indicate metabolism of FAs and thus give us another method to understand the relationship between genetics and FA levels. We also included FA dietary intake and supplement variables and custom-selected for each FA or ratio in our statistical models in order to reduce extraneous variation in red-blood cell FA levels and improve statistical power. The genotypes were re-imputed using the 1000 genomes reference panel thus increasing the number of SNPs under consideration from 2.5 million to 9.3 million common variants (Minor Allele Frequency (MAF)>1%). Finally, we conducted both single-marker tests of gene variant-FA associations and gene-based (multiple-marker) tests.

## Methods

### Sample

Children and spouses of the original Framingham Heart Study cohort were recruited in 1971 and constitute the Framingham Offspring study. These individuals were the focus of our analysis. Detailed descriptions of the sample are available elsewhere [9–12]. Our analysis focused on 2374 individuals who are a subset of 2899 individuals from the offspring cohort who attended Examination 8 between 2005 and 2008, with available genotype data, FA profiles, dietary covariates and who provided consent. Written informed consent was provided by all participants and the Institutional Review Board at Dordt College and the Boston University Medical Center approved the study protocol. The average age of our sample was 66.36 years at time of FA measurement (standard deviation ±8.87), 45.33% of our population is male, 903 families are included in our sample and the average number of people per family is 2.68.

### Fatty acids and ratios

RBC samples were analyzed for glycerophospholipid FA composition using gas chromatography as previously described [1]. In a previous analysis [6], we explored potential genetic associations for 14 FAs (arachidonic acid (AA), dihomo-gamma-linolenic acid (DGLA), docosahexaenoic acid (DHA), docosapentaenoic acid-n3 (DPA-n3), docosapentaenoic acid-n6 (DPA-n6), docosatetraenoic acid (DTA), eicosapentaenoic acid (EPA), linoleic acid (LA), oleic acid (OA), palmitic acid (PA), stearic acid (SA), palmitoleic acid (POA), gamma-linolenic acid (GLA), and alpha-linolenic acid (ALA)). We now expand our analysis to include the eight additional FAs which were measured on this sample (myristic acid (MA), palmitelaidic acid (PLTA), trans oleic acid (TOA), trans linoleic acid (TLA), eicosenoic acid (ESA), eicosadienoic acid (EDA), lingnoceric acid (LNA) and nervonic Acid (NA)). We also considered 15 different ratios of FAs including ratios estimating desaturation, elongation and oxidation processes [8] (see S1 Table for details). Analyses of FA and FA ratios were adjusted for dietary covariates based on daily FA intakes estimated from a Food Frequency Questionnaire [13]. S1 Table provides a complete listing of which FAs or ratios were adjusted for which dietary FA covariates including the specific FAs involved in each FA ratio representing metabolic activity of specific proteins in FA pathways.

### Genetic information

Genotypes were originally measured using both an Affymetrix 500K and a 50K Human Gene Focused Panel as previously described [9]. We imputed these markers on the University of Michigan imputation server using the 1000 genomes Phase 1 imputation, yielding 9.3 million variants meeting standard quality control criteria and with MAF>1% [14]. The Michigan Imputation Server phased our genetic material using Eagle2 [15], as well as provided standard quality control by checking the reference build, duplicate sites, monomorphic sites, and MAF [14]. The imputed SNPs that were used in analysis ranged in value from 0 to 2 with 0 meaning no copies of mutant allele were estimated for the individual while a value of 2 indicates that an individual has 2 copies of the minor allele. Subsequent to imputation, SNPs were assigned to genes using Ensembl release 75, based on GRCh37.p13 [16]. When conducting gene-based tests, all Ensembl genes with location information on one of the 22 autosomes were included in our analysis, for a total of 30,760 genes. SNPs with locations within the gene regions specified in the Ensembl release were included in the gene based analysis.

### Statistical analysis

We first used winsorization to reduce the impact of outliers in FAs and FA ratios more than 5 SDs from the mean after natural log-transformation. The first stage of analysis created residual FA levels by removing the variation in FA levels directly caused by age, sex, relevant dietary covariates (see S1 Table, which assigns each dietary fatty acid to the same red blood cell fatty acid and the other fatty acids derived from it utilizing information from well-known fatty acid pathways) and a kinship matrix summarizing the family structure of the Framingham data using the lmekin function in R [17]. This function implements a linear mixed effects model to predict the fatty acid using the kinship and other covariates. Dietary covariate variables were derived by the FHS using results from the Willet Food Frequency Questionnaire [18] at the time of RBC FA measurement. The residuals from the output of these precursor models were then stored and used as the inputs for all additional modelling steps. By utilizing this two-step method, we were able to drastically reduce the amount of time needed to run this GWAS due to the lengthy computational step of using lmekin to adjust for family structure, which only needed to be done once per FA or FA ratio using this approach.

The 8 FAs and 15 FA ratios which had not yet been analyzed on this sample were analyzed using single-marker testing without dietary covariate adjustment by using the 9.3 million marker genotypes with MAF>1% which passed standard imputation quality control metrics [this GWAS was run in the same method as described in the previous paragraph with the exception of the dietary covariates not being included in the first stage of analysis). For the dietary covariate adjusted analyses, all 22 FAs and 15 FA-ratios were analyzed in a similar manner, but using the precursor models which adjusted for dietary covariates. Subsequently, the GATES method [19], a multiple marker gene-based test, was used to conduct gene-based testing by combining individual SNP-marker p-values. Single-marker tests were deemed statistically significant when the p-value was less than 5x10^-8^ (standard GWAS significance threshold), and gene-based tests were declared significant when p<1.67x10^-6^ (Bonferonni correction for approximately 30,000 gene based tests). LocusZoom [20] was used to generate figures depicting statistical significance, LD structure and gene-locations. Genomic control lambdas (λ_GC_) were estimated using standard median chi square calculations and Q-Q plots were computed using the qqman package in R [17].

## Results

The results of our quality check data indicated that the majority of λ_GC_ values were less than 1.07, and all were less than 1.1, showing little evidence for over-inflation of test-statistics [21].

### Single marker analyses with no dietary covariates

We tested 8 FAs in models without dietary covariates that were not included in our original publication [6], which adjusted only for age, sex and family structure. Two of these FAs (MA and EDA) yielded at least one significant association, with six yielding no significant associations at the 5x10^-8^ significance level. Across the 15 FA ratios tested in models without dietary covariates, ten yielded significant associations (D5D_C20, D6D_C18, D9D_16_18, D9D_C18, ELONG5_N6, ELONG2_N6, D6D+ELONG5(N6;C18), OXD_N3, OXD_N3_N6 and OXD_N6 –see S1 Table for FA involved in each ratio), while five did not (D9D_C16, ELONG2_N3, ELONG2_N3_N6, ELONG6_SAT and ELONG6_MONO). Across all 23 models (8 FA; 15 FA ratios), there were 614 significant FA(ratio)-SNP associations covering 252 unique SNPs and nine unique 1MB or smaller loci on seven different chromosomes. S2 Table has the full listing of all 614 significant SNPs, and S3 Table provides a summarized version of this table by non-overlapping 1MB region and indicates the presence of prior GWAS evidence. We note that these two supplemental tables can be easily sorted by fatty acid instead of by genomic region if interested in particular fatty acid results.

Three of the nine regions identified in S3 Table (Chr 1: *TRIM58*; Chr 6: *ELOVL2*; Chr 11: *FADS* genes) had been identified as associated with different FAs in prior analyses on this sample [6]. The remaining six loci were not identified in prior analyses on this sample.

### SNP and gene-based analyses with dietary covariates

We tested the complete set of 22 FAs and 15 FA ratios in models adjusting for age, sex, family structure and different dietary covariates for each FA or ratio (see S1 Table for full listing of FAs tested and covariates included). After adjusting for dietary covariates, 3143 SNP-FA models were statistically significant (p<5x10^-8^) (see S4 Table). Gene-based tests uncovered 199 significant gene-FA combinations (see S5 Table) at the significance threshold of p<1.67x10^-6^. However, none of the significant gene-based tests identified loci not already identified through SNP-FA tests. Thus, the remaining description of results will be on SNP-FA findings only.

A summary of the eleven distinct, significant 1MB loci identified by SNP-based tests is provided in Table 1. Four of the eleven loci in Table 1 were identified and discussed in prior analyses on this cohort [6]: (Chr 3 (*PCOLCE2*); Chr 6 (*ELOVL2*); Chr 11 (*FADS* complex); Chr 12 (*LPCAT3*)). These results are not summarized in detail here, though some minor differences in which SNPs are significant and at what significance level are present (see S4 Table for full listing), however effect sizes and directions remained the same. We note that S4 Table can be easily sorted by fatty acid instead of by genomic region if interested in particular fatty acid results.

**Table 1:** Summary of 11 significant loci for FAs and FA ratios with dietary covariates.

Chr	Size of region (kb)	Location (kb)	# sig. SNPs^1^	Genes^2^	Prior GWAS evidence^3^	Evidence without dietary covariates^4^	Smallest p-value	SNP ID^5^	Fatty acid or FA ratio^5^^,^^6^
3	23	142631	7	PCOLCE2	Yes	Yes	2x10^-9^	rs2619150	AA
6	290	10947	290	SYCP2L, ELOVL2	Yes	Yes	1.45 x 10^−19^	rs3734398	DHA_DPAn3
6	8	135402	11	HBS1L	Yes	Yes	3.4x10^-9^	rs1331309	DTA_AA
6	58	161641	6	AGPAT4	Yes	Yes	2.4x10^-17^	rs75534358	DTA_AA
7	12	71434	4	CALN1	Yes	No	3.1x10^-8^	rs35928775	EPA
10	1	102075	3	PKD2L1	Yes	No	3.6x10^-9^	rs603424	POA_PA
11	1	37700	1	none	No	No	4.6x10^-8^	rs1461903	DHA_DPAN3
11	572	61296	2367	SYT7, RPLPOP2, DAGLA, MYRF, MIR611, FADS1, MIR1908, FADS2, FADS3, MIR6746, RAB3IL1, FTH1, BEST1, FEN1, TMEM258	Yes	Yes	5.8x10^-244^	rs174544	AA_DGLA
12	294	6980	332	MIR200C, EMG1, C1S, LPCAT3, SCARNA12, PHB2, PTPN6, MIR141	Yes	Yes	5.5 x 10^−43^	rs73264687	OA
14	1	24462	1	DHRS4L2	Yes	No	4.3x10^-8^	rs111387220	POA_PA_GLA_LA
16	348	15052	122	PDXDC1; NTAN1	Yes	Yes	1.3 x 10^−13^	rs4985155	DGLA_LA

1 All 3143 significant SNP-FA combinations are provided in S4 Table.

2 All genetic data based on genome build grch37

3 Based on searches at www.ebi.ac.uk/gwas

4 Based on our prior analysis of 14 FAs on this sample in Tintle et al. (2015) or 22 other FAs/FA-ratios as presented in S3 Table

5 SNP ID and FA/FA ratio are specific to the model with the smallest p-value in the specified chromosome region.

6 Lists of dietary covariates for each fatty acid or ratio are provided in S1 Table

Of the remaining seven loci in Table 1, three were also identified in models which did not adjust for dietary covariates (Chr 6 (*HBS1L* with ELONG2_N6); Chr 6 (*AGAPT4* with ELONG2_N6); Chr 16 (*PDXDC1* with D6D+ELONG5(N6;C18))), while four were only significant in models adjusting for covariates (Chr 7 (*CALN1* with EPA); Chr 10 (*PKD2L1* with D9D_C16); Chr 11 (rs1461903 with OXD_N3); Chr 14 (*DHRS4L2* with D9D_16_18)). The pattern of results from these seven loci [and from two other loci from S3 Table (Chr 2 (*RAB3GAP1/MCM6*); Chr 15 (LOC102723481) which were no longer significant after dietary covariate adjustment), are briefly described in the sections below. Manhattan plots are available as supplemental figures (S1–S9 Manhattan Plots described in Supporting Information subsection) for the results described in the subsequent sections.

#### Chromosome 2 –*RAB3GAP1/MCM6*

Twenty-three SNPs on chromosome 2 in the *RAB3GAP1/MCM6* region were significantly associated with D9D_16_18 (a ratio of ratios: POA:PA to GLA:LA; representing the relative desaturation efficiency of 16- versus 18-carbon FAs) levels. The twenty-three identified SNPs have similar effect allele frequencies (EAFs) (22–24%) and similar beta values (approximately 3% decrease with each additional effect allele), indicating lower levels of the D9D_16_18 ratio with additional copies of the less frequent allele, and implying a loss of efficiency in desaturating 16-carbon FAs or a gain in efficiency on 18-carbon FAs. These SNPs were no longer significant at the genome-wide level after adjustment for dietary ratios of the same FAs indicating that the polymorphism effects on FA levels may be mediated by intake.

#### Chromosome 6 –*HBS1L*

**Fig 1:**
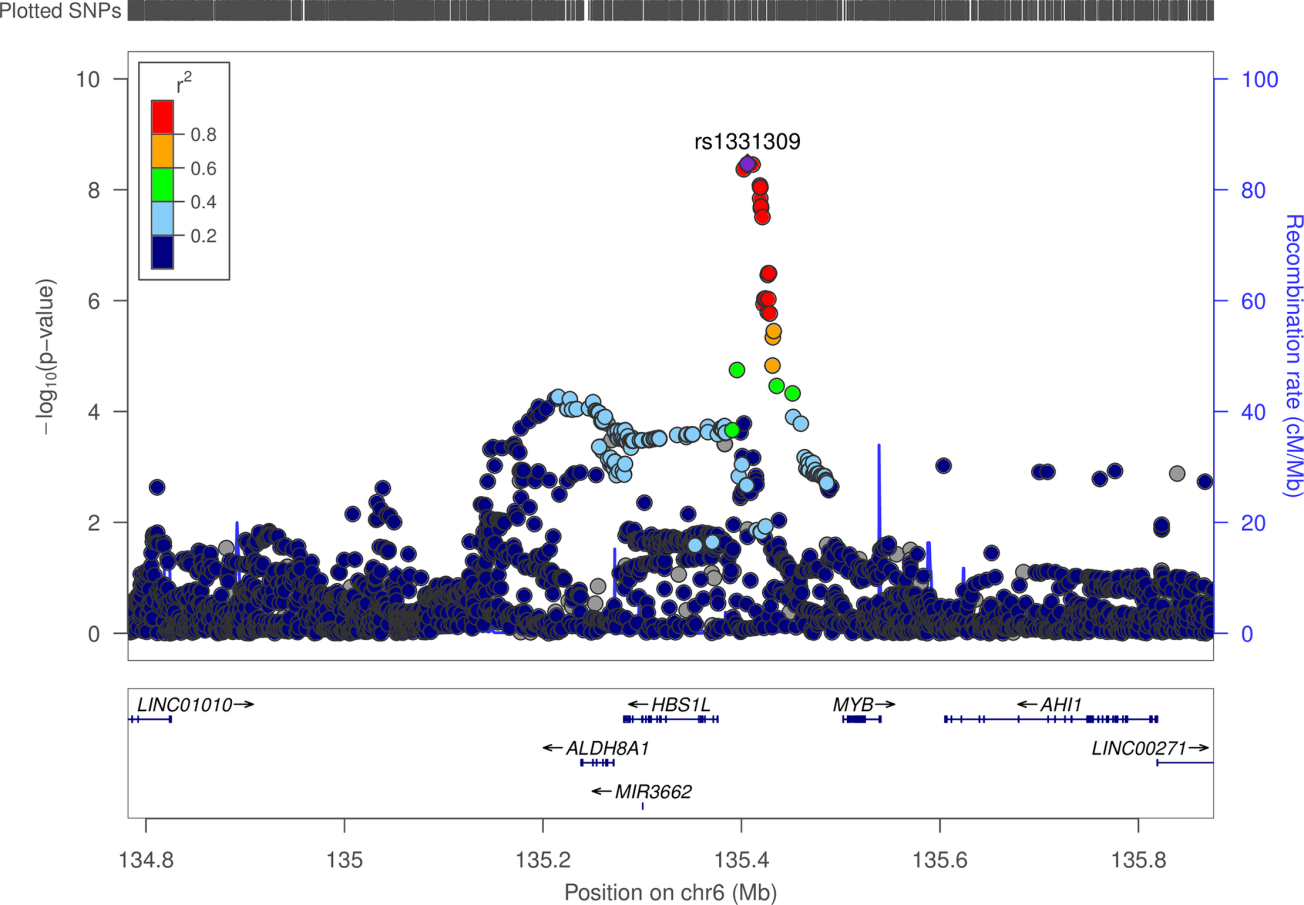
LD structure for variants associated with ELONG2_N6 in and near *HBS1L*.

Eleven SNPs on chromosome 6 in the *HBS1L* (bp 135,281,516 to 135,424,194) gene were significantly associated with ELONG2_N6 (DTA to AA) levels. All eleven SNPs had similar EAFs (approximately 24%). The p-values ranged from 3.1x10^-8^ to 3.4x10^-9^ (see Fig 1). The less frequent alleles for all of the significant SNPs were associated with higher ELONG2_N6 levels (1.29% increase/allele) consistent with greater elongation activity on arachidonate. This locus was also identified prior to adjusting for dietary covariates (S3 Table).

#### Chromosome 6 –*AGPAT4*

**Fig 2:**
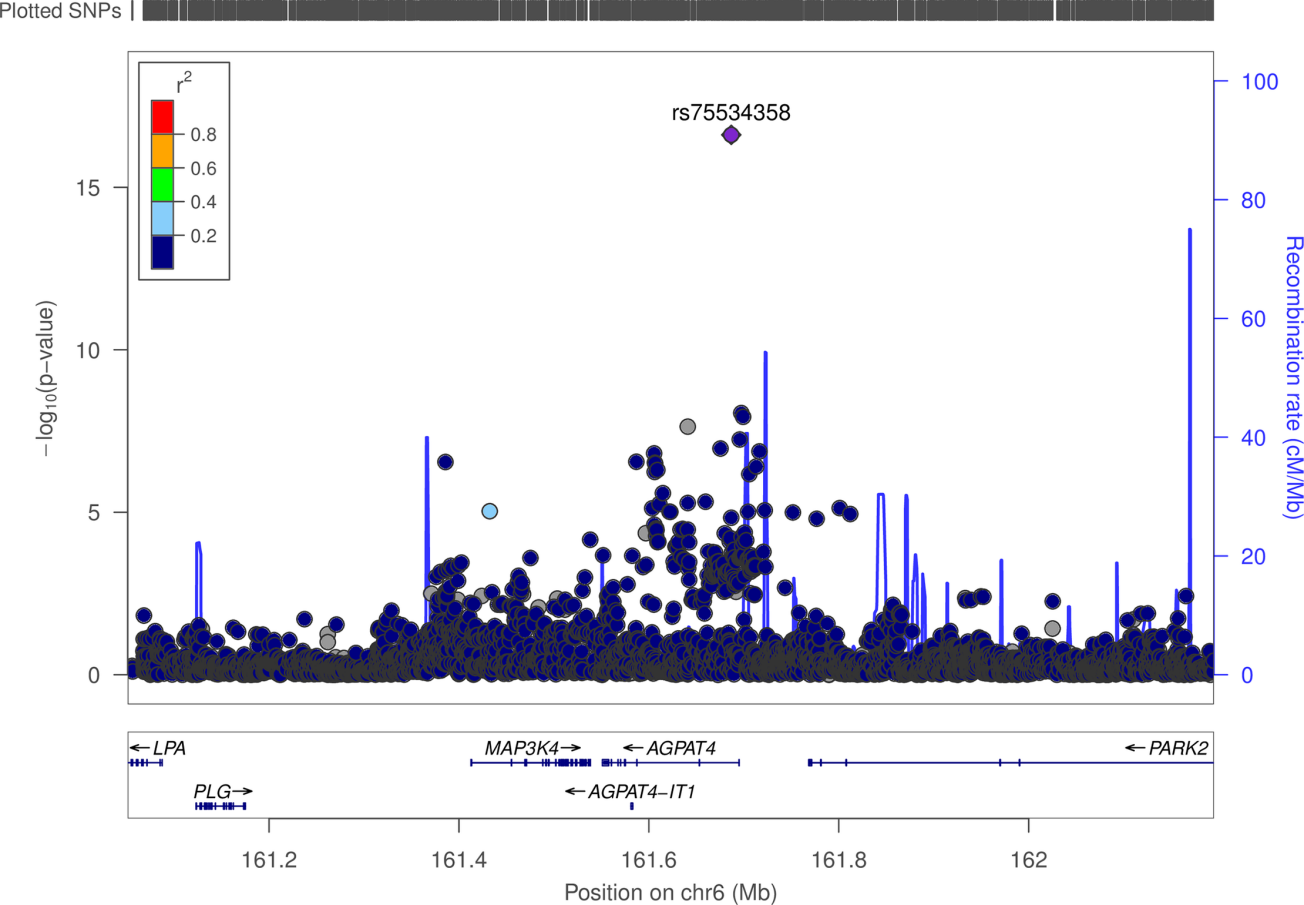
LD structure for variants associated with ELONG2_N6 in and near *AGPAT4*.

Another region on chromosome 6, gene *AGPAT4* (bp 161,551,011 to 161,695,093), also showed both positive and negative association with ELONG2_N6 (DTA to AA) levels as well as associations with DTA and ELONG2_N3_N6 levels (DPA_N3: EPA to DTA: AA). One SNP (rs75534358) is associated with all three biomarkers of FA levels, the most significant being ELONG2_N6 (p-value: 2.43x10^-17^) with an effect size of -5.72%. However, the other 3 SNPs associated with ELONG2_N6 show evidence of effects in the opposite direction of the first SNP. Two of these SNPs (upstream of *AGPAT4*) had dramatically different EAFs than rs75534358 (34%, and 3.4%, respectively), while the third SNP (downstream of *AGPAT4*) was similar (1.5%). See Fig 2 for an illustration of the LD structure.

#### Chromosome 7 –*CALN1*

**Fig 3:**
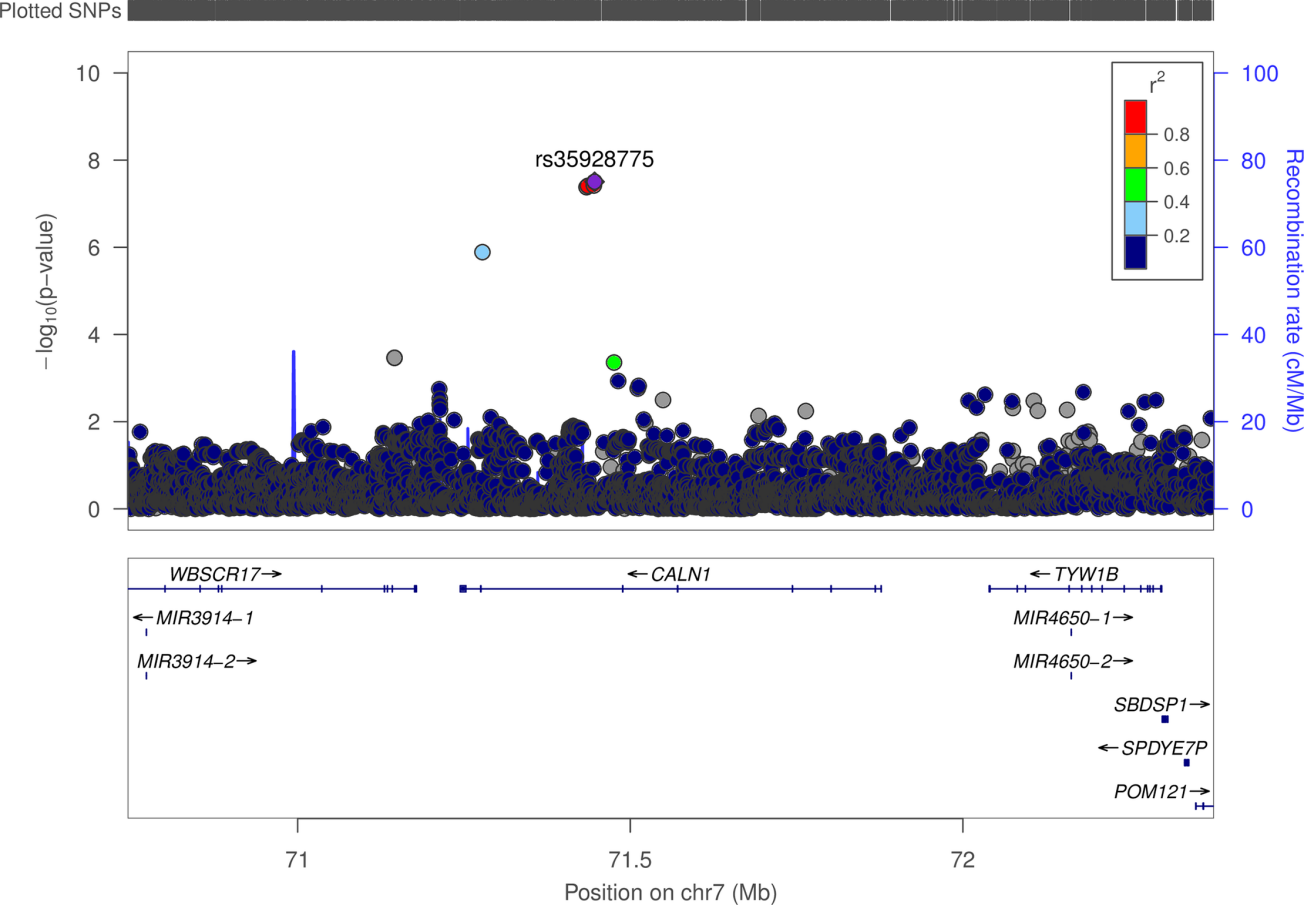
LD structure for variants associated with EPA in and near *CALN1*.

Chromosome 7 has four SNPs associated with EPA. These SNPs are in the *CALN1* gene which spans from bp 71,244,476 to 71,912,136. The p-values range from 4.17x10^-8^ to 3.14x10^-8^ with an average EAF of approximately 2.0%. All four SNPs are associated with higher levels of EPA (average effect size: 38.8%). Fig 3 highlights the pattern of significance and LD structure for this gene.

#### Chromosome 10 –*PKD2L1*

One SNP (rs603424) in the *PKD2L1* gene (bp 102,047,903 to 102,090,243) on chromosome 10 is significantly associated with ELONG6_MONO (OA to POA), POA, and D9D_16_18 (POA:PA to GLA:LA). The most significant association with a p-value of 3.56x10^-9^ is with D9D_16_18. The SNP is associated with higher levels of ELONG6_MONO (effect size: 7.16%) and lower levels of POA and D9D_16_18 (effect size: -7.64%). The EAF for all three associations is 23%.

#### Chromosome 11– rs1461903

One SNP in chromosome 11 at bp 37,700,125 is associated with OXD_N3 (DHA to DPA_N3) with a p-value of 4.58x10^-8^. This SNP is more than 23MB from the *FADS* gene area, which houses over two-thousand SNPs. The effect size of this SNP is -2.93% and the EAF is 42%. There are no known genes near this SNP.

#### Chromosome 14 –*DHRS4L2*

**Fig 4:**
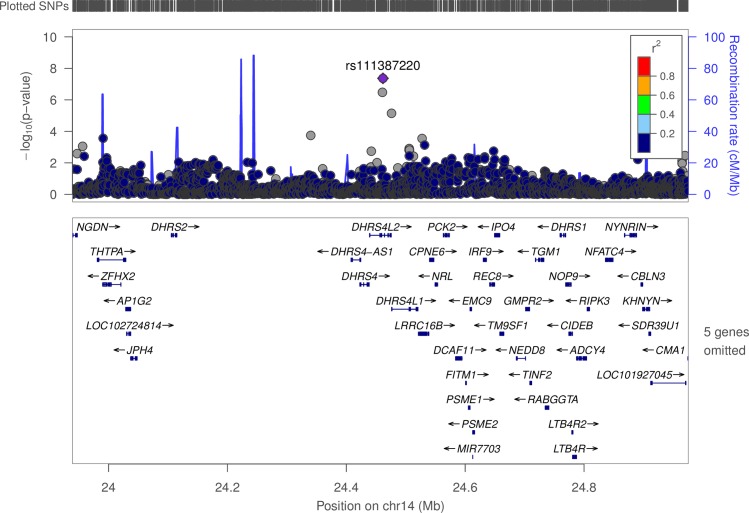
LD structure for variants associated with D9D_16_18 in and near *DHRS4L2*.

In the gene *DHRS4L2*, which runs from bp 24,439,148 to 24,475,617, there is one significant SNP (rs111387220) that is associated with the D9D_16_18 (POA:PA to GLA:LA) FA ratio. This SNP is associated with lower levels (effect size: -99.1%) of the FA ratio with a p-value of 4.31x10^-8^, thus indicating that this SNP correlates with less efficient desaturation of 16-carbon FAs compared to 18-carbon FAs. The EAF is 1.7%. Fig 4 illustrates significance and LD structure in and around this gene.

#### Chromosome 15 –LOC102723481

Two SNPs were associated with myristic acid levels (effect size: -4.09%), such that additional copies of the less frequent allele were associated with lower myristic acid levels. These SNPs were no longer significant after adjusting for dietary covariates indicating that the polymorphism effects on FA levels are mediated by intake.

#### Chromosome 16 –*NTAN1*, *PDXDC1*

122 SNPs on Chromosome 16 within genes *NTAN1* and *PDXDC1* showed significant association with D6D+ELONG5(N6;C18) (DGLA: LA) as well as D9D_16_18 (POA:PA to GLA:LA). The most significant SNP (p-value of 1.31x10^-13^) was rs4985155 which is associated with lower levels of D6D+ELONG5(N6;C18). These variants had EAFs between 7.4% and 6.5%, with effect sizes between -20.90% and 21.67%.

## Discussion

In previous analyses on this data set, which examined a limited group of FAs without consideration of dietary covariates, we identified five loci associated with various FA levels [6]. These five loci included the *FADS* gene complex and the *ELOVL2* gene which have been repeatedly identified as directly related to the desaturation and elongation of FAs, respectively [22,23]. In our previous analysis the *PCOLCE2* gene and *LPCAT3* genes were also identified. Recent biological evidence suggests strong causal links between *PCOLCE2* activity and atherosclerosis [24], while a series of recent articles are beginning to tease out the phospholipid related functionality of *LPCAT3* [25–29]. *LPCAT3* was also recently found to be associated with FA in a separate GWAS [30]. *TRIM58*, which we identified earlier in this population when not adjusting for dietary covariates, was no longer significant after adjusting for dietary covariates, suggesting that diet may mediate the effects of *TRIM58*; this idea has only begun to be explored in the literature [31].

Notably, we detected four new loci after adjusting for dietary covariates, which did not reach genome-wide significance before dietary covariate adjustment. These findings also demonstrate general similarities to our previous GWAS by identifying nine of the same loci after dietary covariate adjustment. Thus, the impact of adjusting for dietary covariates in GWAS of FAs and FA ratios may be minimal. However, we note that while the use of self-reported dietary intake via a food-frequency questionnaire is standard practice, the substantial variability in measurements provided by this approach may limit the impact of these covariates in subsequent models attempting to identify true gene-FA relationships.

We also note that gene-based testing using the GATES approach to combine single-marker common variant p-values detected no novel loci as statistically significant which weren’t already identified as statistically significant in single marker tests despite using a less stringent multiple testing threshold. Multi-marker tests like GATES are optimized for detecting loci with multiple, independent effects on phenotypes of interest. The lack of novel, significant findings using GATES suggests that many of the findings here may represent a single association with FAs or FA ratios at each locus, though further analysis is necessary.

Our focus on common variants (vs. rare), linear relationships (vs. non-linear) between genetic variations and FA-levels and lack of consideration of gene-environment or gene-gene interactions suggest that numerous other models are worth exploring in subsequent analyses. These may be particularly fruitful and necessary given the relatively low explanatory power of currently known genetic variants at explaining FA levels.

We focus the remainder of our discussion on nine loci, which we did not identify in prior analyses using this data set.

### Chromosome 2 (*RAB3GAP1/MCM6*)

In earlier analyses of this sample, which did not consider FA ratios, we did not identify this region as having significantly associated SNPs. However, analyses on other independent samples have identified SNPs and genes in this region as associated with saturated and monounsaturated FA plasma levels [4], phospholipid levels [2,32], cholesterol [33,34], blood metabolites [35], body mass index [36] and cardiac death [37]. *RAB3GAP1* is strongly associated with microcephaly, consistent with aberrations in FA trafficking to the brain, the organ most associated with structural use of FAs, and a function likely to be affected by altered discrimination of FAs by chain length. *MCM6* is most strongly associated with lactase deficiency [38,39], an enzyme critical to digestion of milk. Milk is an abundant source of the 16-carbon PA, and thus behavioral adjustment to this deficiency could alter the availability of dietary FAs.

### Chromosome 6 (*HBS1L/MYB*)

Our analysis found evidence of association between the ELONG2_N6 and variants in the *HBS1L* gene. Prior GWAS have identified variants in *HBS1L* as associated with red blood cell [40] and platelet [41] phenotypes. A recent GWAS also identified association between DTA levels and MYB variants [3]. *HBS1L* is a determinant of the final stages of erythropoiesis, and is associated with β-thalassemia [42]. Simple alterations in erythrocyte maturation is an eminently plausible explanation for this association, however, the role of signaling metabolite derived from arachidonate should also be considered.

### Chromosome 6 (*AGPAT4*)

We identified four variants in *AGPAT4* associated with DTA and related ratios. This finding supports another recent GWAS result which found evidence of *AGPAT4* association with DTA levels in a trans-ethnic meta-analysis study [3], and is known to encode a key step in phospholipid synthesis [43].

### Chromosome 7 (*CALN1*)

Our analysis identified somewhat infrequent occurring variants (EAF of ~2%) in *CALN1* as associated with EPA levels. While previous GWAS have identified variants in *CALN1* as associated with lipid levels [33,34] and schizophrenia [44], no prior GWAS had found associations between *CALN1* variants and FA levels. While this gene is relatively under-studied, a recent study has hypothesized that a micro-RNA (MIR137, also associated with schizophrenia risk) targets the *CALN1* gene [45]. Given the association between EPA and schizophrenia [46–48], this locus may be a valuable source for understanding the role of EPA in mental health.

### Chromosome 10 (*PKD2L1*)

Our analysis found significant evidence of association between POA levels (and two ratios involving POA: D9D_C16 and ELONG6_MONO) with a single SNP variant in *PKD2L1* (a polycystin protein gene). A recent GWAS found evidence of association with SNPs in this gene and palmitoleic acid levels [4].

### Chromosome 11 (rs1461903)

This single SNP which we found associated with the OXD_N3 has not been identified in prior GWAS. It does not sit in a gene (the nearest is a pseudo-gene approximately 79kb away: *RPL7AP56*).

### Chromosome 14 (*DHRS4L2*)

We identified a single SNP associated with the ratio of D9D_16_18 levels in the gene *DHRS4L2* with relatively infrequent occurrence of the mutated allele (1.74%). One prior GWAS identified this gene as associated with bi-polar disorder [49]. The gene encodes a short-chain dehydrogenase enzyme, which have broad dehydrogenase activities on lipid substrates [50].

### Chromosome 15 (LOC102723481)

We identified the uncharacterized protein LOC102723481 as associated with myristic acid levels. While this region has received little attention in the literature, one recent GWAS [51] did find associations with smoking/lung cancer. Prior work has hypothesized a relationship between smoking and myristic acid levels [51], which could be suggestive of a biological mechanism for this finding.

### Chromosome 16 (*NTAN1/PDXDC1*)

We found evidence of association in the activity of D6D+ELONG5(N6;C18) and D9D_16_18, with variants in *NTAN1/PDXDC1*. Prior GWAS have also identified variants in this region as separately associated with both LA and DGLA levels [52], interactions between dietary FAs and genetic variants on tissue FA levels [5], ALA levels [2], four FAs (LA, GLA, DGAL and AA) [3], as well as lysophosphatidylcholines and phosphatidylcholines [32]. Recent wet-lab experimentation has verified changes in proteins related to *PDXDC1* levels in mice which were fed a high fat diet [53]. *NTAN1* has also been associated with schizophrenia [54], repeating a broader pattern of convergence between tissue FAs and cognitive function.

## Limitations

There are a number of limitations of our current analysis which are worth noting here. First, power in genome-wide association studies continues to be a challenge faced by many studies, and the analysis here is no different. While we analyzed the maximum amount of samples for which we had access, and sample sizes are in line with many previous publications, future studies with larger sample sizes will be necessary to replicate the findings of this study in independent samples. These challenges are further compounded by the necessarily complex correlation structure between fatty acids. While we have analyzed fatty acids and ratios separately here, future studies may wish to consider the use of methods that explicitly consider the correlation structure of fatty acids in order to more carefully elucidate fatty acids directly impacted by genetic variation. It is also worth mentioning that while we have controlled for dietary fatty acid intake, this data is based on self-report and not a well-controlled instrument. The additional variability, and potential bias, from the use of self-reported dietary information, is likely further contributing to power concerns. However, the food frequency questionnaire used here remains an industry standard and few widely used, valid and reliable alternatives exist. Finally, we note that the focus of our analysis in this GWAS framework is association vs. causal, and additional data (e.g., sequencing) and analyses (e.g., Mendelian randomization) will be necessary to more specifically identify causal variants at the locus, including identification of whether there are multiple causal variants within a particular gene of interest.

## Conclusions

This analysis identifies loci previously reported in other genome-wide studies, while identifying four novel loci and nine others previously identified by other GWAS on this population, in the search for genes which directly contribute to variation in fatty-acid levels. Wet-lab exploration of these novel loci will likely contribute greatly to the understanding of their role in FA metabolism and synthesis. Given the relatively small sample size of our study and examination of multiple FAs and FA ratios, future work should also likely include meta-analyses to examine larger sample sizes and sequencing data to explore the potential contribution of rare variants towards variation in FA levels.

## Supporting information

S1 Table Table of FAs, FA ratios, dataset variable names for FAs/FA ratios, and the significant dietary covariates for each FA/FA ratio. (DOCX)

S2 Table Significant results from the GWAS predicting FA (8) and FA ratios (15) without dietary covariates. (CSV)

S3 Table Condensed version of S2 Table SNP results into 1 megabase regions. (DOCX)

S4 Table Significant results from the GWAS predicting FA and FA ratios adjusted for dietary covariates. (CSV)

S5 Table Significant results of the gene based tests that predicted FA/FA ratios using SNP data including adjustment for dietary covariates. (XLSX)

S1 Manhattan Plot Manhattan plot of DGLA:LA GWAS results. (PNG)

S2 Manhattan Plot Manhattan plot of DHA:DPAN3 GWAS results. (PNG)

S3 Manhattan Plot Manhattan plot of DPAN3:EPA to DTA:AA GWAS results. (PNG)

S4 Manhattan Plot Manhattan plot of DTA:AA GWAS results. (PNG)

S5 Manhattan Plot Manhattan plot of EDA GWAS results. (PNG)

S6 Manhattan Plot Manhattan plot of EPA GWAS results. (PNG)

S7 Manhattan Plot Manhattan plot of MA GWAS results. (PNG)

S8 Manhattan Plot Manhattan plot of OA:POA GWAS results. (PNG)

S9 Manhattan Plot Manhattan plot of POA:PA to GLA:LA GWAS results. (PNG)
