# Supplementary material for: A genome-wide association study of red-blood cell fatty acids and ratios incorporating dietary covariates: Framingham Heart Study Offspring Cohort
Source: PLoS One. 2018 Apr 13;13(4):e0194882. doi: 10.1371/journal.pone.0194882 (PMC5898718; doi:10.1371/journal.pone.0194882)
Supplement: S1 Table — (DOCX) [file pone.0194882.s001.docx]

**Supplemental Table 1. Covariate adjustments for fatty acid GWAS analysis**

| Fatty acid or ratio (abbreviation) | SHARE Variable Name  or Ratio Calculation | Dietary Model Covariates^2^ |
| --- | --- | --- |
| INDIVIDUAL FATTY ACIDS | | |
| Palmitic Acid (PA)^1^ | RBC_C16_0 | NUT_F160 |
| Stearic Acid (SA) ^1^ | RBC_C18_0 | NUT_F180 |
| Palmitoleic Acid (POA) ^1^ | RBC_C16_1 | NUT_F160, NUT_F161 |
| Oleic Acid (OA) ^1^ | RBC_C18_1 | NUT_F180, NUT_F181 |
| Eicosapentaenoic Acid (EPA) ^1^ | RBC_C20_5N3 | NUT_F183, NUT_F205, FishOil |
| Docosapentaenoic Acid n-3 (DPA_N3) ^1^ | RBC_C22_5N3 | NUT_F183, NUT_F205, NUT_F225, FishOil |
| Docosahexaenoic Acid (DHA) ^1^ | RBC_C22_6N3 | NUT_F183, NUT_F205, NUT_F225, NUT_F226, FishOil |
| Alpha-linolenic Acid (ALA) ^1^ | RBC_C18_3N3 | NUT_F183 |
| Linoleic Acid (LA) ^1^ | RBC_C18_2N6 | NUT_F182 |
| Gamma-linolenic Acid (GLA) ^1^ | RBC_C18_3N6 | NUT_F182 |
| Dihomo-gamma-linoleic Acid (DGLA) ^1^ | RBC_C20_3N6 | NUT_F182 |
| Arachidonic Acid (AA) ^1^ | RBC_C20_4N6 | NUT_F182, NUT_F204, NUT_F160, NUT_F205, NUT_F226, FishOil |
| Docosapentaenoic Acid-n6 (DPA_N6) ^1^ | RBC_C22_5N6 | NUT_F204, NUT_F226, FishOil |
| Docosatetranoic Acid (DTA) ^1^ | RBC_C22_4N6 | NUT_F204 |
| Myristic Acid (MA) | RBC_C14_0 | NUT_F140 |
| Palmitelaidic Acid (PLTA) | RBC_C16_1t | NUT_T161 |
| Trans oleic Acid (TOA) | RBC_C18_1t | NUT_T181 |
| Trans linoleic Acid (TLA) | RBC_C18_2t | NUT_T182 |
| Eicosenoic Acid (ESA) | RBC_C20_1 | NUT_F201 |
| Eicosadienoic Acid (EDA) | RBC_C20_2n6 | None |
| Lingnoceric Acid (LNA) | RBC_C24_0 | None |
| Nervonic Acid (NA) | RBC_C24_1 | None |
| DESATURATION PROCESSES | | |
| AA:DGLA (D5D_C20) | RBC_C20_4N6 / RBC_C20_3N6 | NUT_F182, NUT_F204, NUT_F160, NUT_F205, NUT_F226, FishOil |
| GLA: LA (D6D_C18) | RBC_C18_3N6 / RBC_C18_2N6 | NUT_F182 |
| POA: PA (D9D_C16) | RBC_C16_1 / RBC_C16_0 | NUT_F160, NUT_F161 |
| OA:SA (D9D_C18) | RBC_C18_1 / RBC_C18_0 | NUT_F180, NUT_F181 |
| POA:PA::GLA:LA (D9D_16_18) | D9D_C16 / D9D_C18 | NUT_F160, NUT_F161, NUT_F180, NUT_F181 |
| ELONGATION PROCESSES | | |
| DPA_n3: EPA (ELONG2_N3) | RBC_C22_5N3 / RBC_C20_5N3 | NUT_F183, NUT_F205, NUT_F225, FishOil |
| DTA: AA (ELONG2_N6) | RBC_C22_4N6 / RBC_C20_4N6 | NUT_F182, NUT_F204, NUT_F160, NUT_F205, NUT_F226, FishOil |
| DPA_n3:EPA::DTA:AA (ELONG2_N3_N6) | ELONG2_N3 / ELONG2_N6 | NUT_F182, NUT_F204, NUT_F160, NUT_F183, NUT_F205, NUT_F225, NUT_F226, FishOil |
| DGLA:GLA (ELONG5_N6) | RBC_C20_3N6/RBC_C18_3N6 | NUT_F182 |
| DGLA: LA (D6D+ELONG5(N6;C18)) | RBC_C20_3N6 / RBC_C18_2N6 | NUT_F182 |
| SA: PA (ELONG6_SAT) | RBC_C18_0 / RBC_C16_0 | NUT_F160, NUT_F180 |
| OA: POA (ELONG6_MONO) | RBC_C18_1 / RBC_C16_1 | NUT_F160, NUT_F161, NUT_F180, NUT_F181 |
| OXIDATION PROCESSES | | |
| DHA: DPA_N3 (OXD_N3) | RBC_C22_6N3 / RBC_C22_5N3 | NUT_F183, NUT_F205, NUT_F225, NUT_F226, FishOil |
| DPA_N6: AA (OXD_N6) | RBC_C22_5N6 / RBC_C22_4N6 | NUT_F204, NUT_F226, FishOil |
| DHA:DPA_N3::DPA_N6:AA (OXD_N3_N6) | OXD_N3 / OXD_N6 | NUT_F183, NUT_F205, NUT_F225, NUT_F226, NUT_F204, FishOil |

1. Analysis without dietary covariates for these fatty acids (age and sex adjusted only) are reported in Tintle et al. (2015)
2. NUT_XXX indicates variable names in dbGaP which are derived based on results of the Food Frequency Questionnaire. Common names of these dietary fatty acids are:
   NUT_F140=Myristic acid
   NUT_F160=Palmitic acid
   NUT_F161=Palmitoleic acid
   NUT_F180=Stearic acid
   NUT_F181=Oleic acid
   NUT_F182=Linoleic acid
   NUT_F183=Linolenic acid
   NUT_F201=Eicosenoic acid
   NUT_F204=Arachadonic acid
   NUT_F205=Eicosapentaenoic acid
   NUT_F225=Docosapentaenoic acid
   NUT_F226=Docosahexaenoic acid
   NUT_T161=Trans Palmitelaidic acid
   NUT_T181=Trans oleic acid
   NUT_T182=Trans linoleic acid
   FishOil indicates taking a cod liver oil pill (CODLIV) or taking an omega-3 supplement (OMEGA3)
