# Supplementary material for: A genome-wide association study of red-blood cell fatty acids and ratios incorporating dietary covariates: Framingham Heart Study Offspring Cohort
Source: PLoS One. 2018 Apr 13;13(4):e0194882. doi: 10.1371/journal.pone.0194882 (PMC5898718; doi:10.1371/journal.pone.0194882)
Supplement: S3 Table — (DOCX) [file pone.0194882.s003.docx]

**Supplemental Table 3. Summary of significant loci for FAs and FA Ratios without Dietary Covariates**

| Chr | Size of region (kb) | Location (kb) | # sig. SNPs^1^ | Genes^2^ | Prior GWAS evidence?^3^ | Evidence from other FAs on this sample^4^ | Smallest p-value | SNP ID^5^ | Fatty acid or FA ratio^5^ |
| --- | --- | --- | --- | --- | --- | --- | --- | --- | --- |
| 1 | 1 | 248039 | 1 | TRIM58 | Yes | Yes | 1x10^-9^ | rs3811444 | D9D_C18 |
| 2 | 987 | 136825-135837 | 23 | RAB3GAP1, ZRANB3, R3HDM1, UBXN4, MCM6, DARS | Yes | No | 4.1x10^-10^ | rs6716536 | D9D_16_18 |
| 6 | 133 | 110845-109517 | 60 | SYCP2L, ELOVL2 | Yes | Yes | 4.4x10^-17^ | rs4711146 | OXD_N3 |
| 6 | 8 | 135419-135411 | 5 | HBS1L | Yes | No | 3.1x10^-9^ | rs9376090 | ELONG2_N6 |
| 6 | 1 | 161606 | 1 | AGPAT4 | Yes | No | 3.6x10^-8^ | rs2064720 | ELONG2_N6 |
| 11 | 488 | 61851-61363 | 496 | RPLP0P2, DAGLA, MYRF, MIR611, FADS1, MIR1908, FADS2, FADS3, MIR6746, RAB3IL1, FTH1 | Yes | Yes | 3.8x10^-247^ | rs968567 | D5D_C20 |
| 12 | 76 | 7146-7070 | 10 | MIR200C, EMG1, C1S | Yes | Yes | 4.3x10^-22^ | rs2110073 | D9D_C18 |
| 15 | 1 | 67230-67231 | 2 | LOC102723481 | No | No | 1.7x10^-8^ | rs16950375 | RBC_C14_0 |
| 16 | 1 | 15129-15129 | 16 | PDXDC1 | Yes | No | 3.0x10^-16^ | rs4985155 | ELONG5_N6 |

1. Genes containing or within 10kb of significant SNPs
2. All 614 significant SNP-FA combinations are provided in Supplemental Table 2
3. Based on searches at http://www.ebi.ac.uk/gwas/location to EBI; not including Tintle et al. (2015)
4. Based on our prior analysis of 14 FAs on this sample in Tintle et al. (2015)
5. SNP ID and FA/FA ratio are specific to the model with the smallest p-value in the specified chromosome region

# All genetic data based on genome build grch37
