# Supplementary figures and images for: A genome-wide association study of red-blood cell fatty acids and ratios incorporating dietary covariates: Framingham Heart Study Offspring Cohort
Source: PLoS One. 2018 Apr 13;13(4):e0194882. doi: 10.1371/journal.pone.0194882 (PMC5898718; doi:10.1371/journal.pone.0194882)

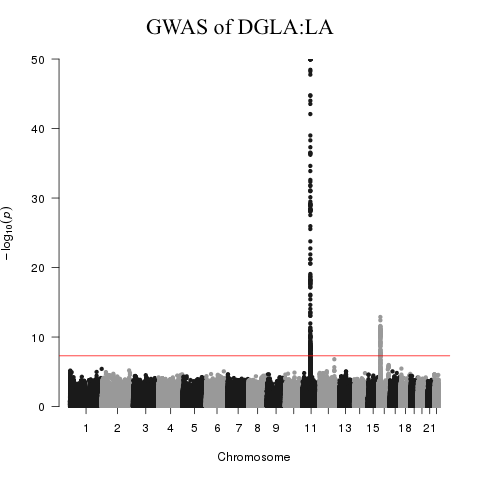

Supplement: S1 Manhattan Plot — (PNG) [file pone.0194882.s006.png]

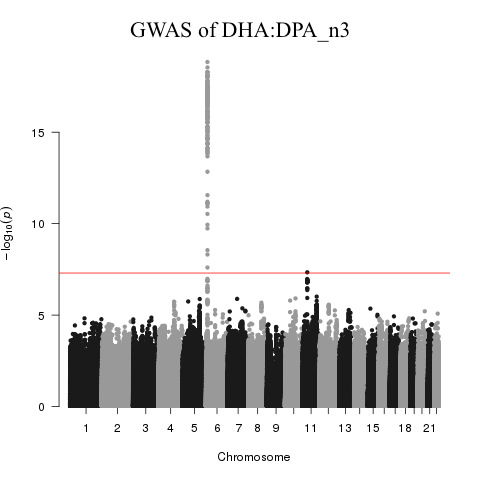

Supplement: S2 Manhattan Plot — (PNG) [file pone.0194882.s007.png]

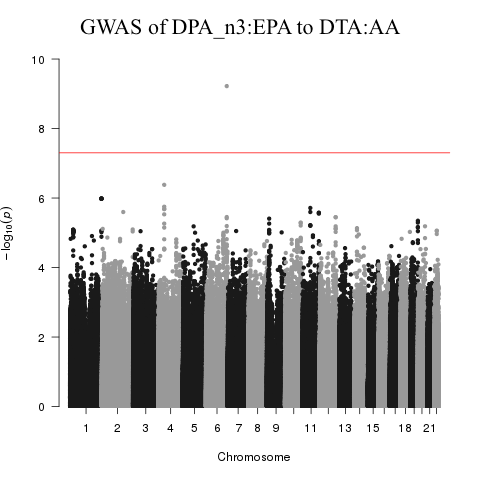

Supplement: S3 Manhattan Plot — (PNG) [file pone.0194882.s008.png]

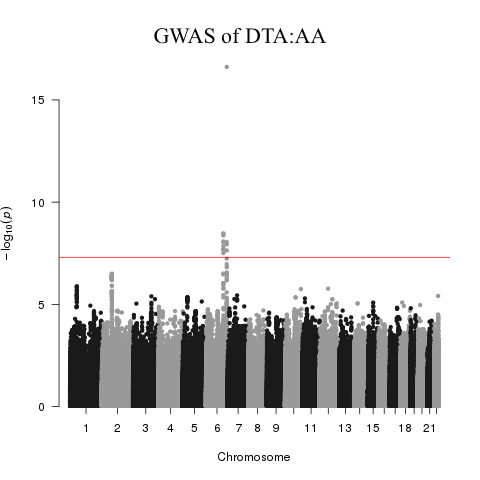

Supplement: S4 Manhattan Plot — (PNG) [file pone.0194882.s009.png]

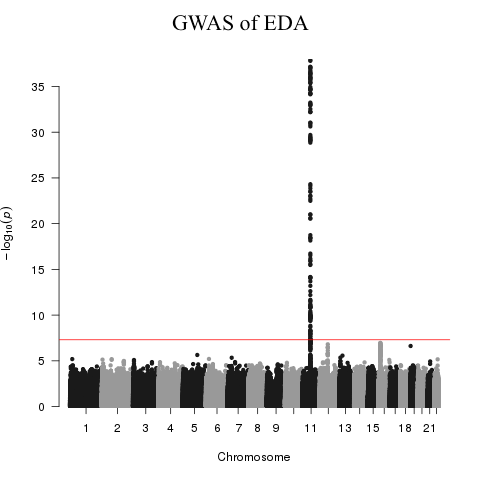

Supplement: S5 Manhattan Plot — (PNG) [file pone.0194882.s010.png]

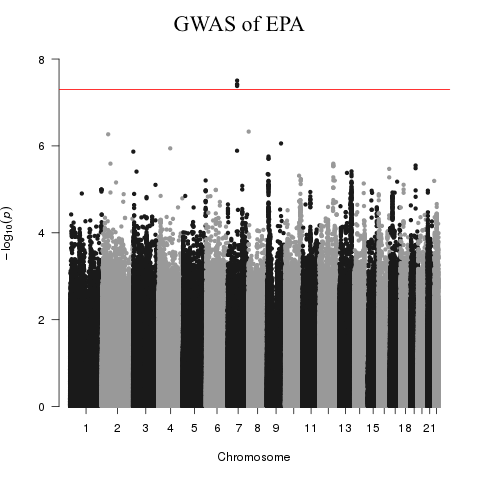

Supplement: S6 Manhattan Plot — (PNG) [file pone.0194882.s011.png]

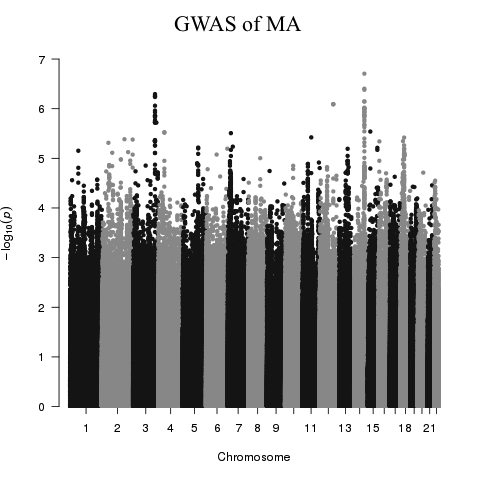

Supplement: S7 Manhattan Plot — (PNG) [file pone.0194882.s012.png]

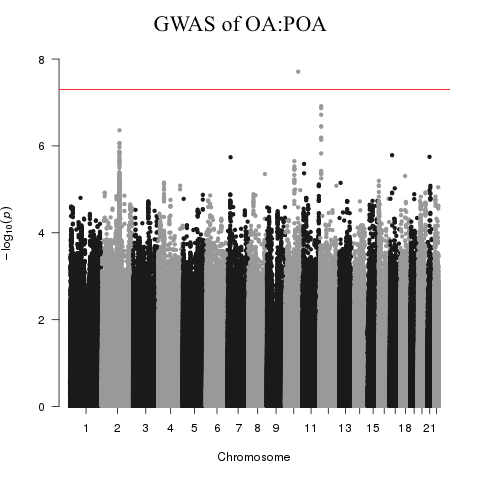

Supplement: S8 Manhattan Plot — (PNG) [file pone.0194882.s013.png]

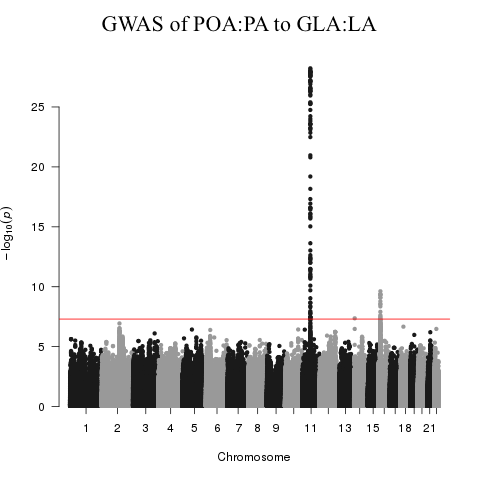

Supplement: S9 Manhattan Plot — (PNG) [file pone.0194882.s014.png]
